# Superselective Catheter Angiographies of the Wrist (SCAW): Approaches for Vascularized Bone Grafts

**DOI:** 10.3390/diagnostics13061198

**Published:** 2023-03-22

**Authors:** Leonie Goelz, Simon Kim, Andreas Eisenschenk, Sven Mutze, Ariane Asmus

**Affiliations:** 1 Department of Radiology and Neuroradiology, BG Klinikum Unfallkrankenhaus Berlin, Warener Str. 7, 12683 Berlin, Germany; 2 Institute for Diagnostic Radiology and Neuroradiology, University Medicine Greifswald, Ferdinand-Sauerbruch-Str., 17475 Greifswald, Germany; 3 Department of Hand Surgery and Microsurgery, University Medicine Greifswald, Ferdinand-Sauerbruch-Str., 17475 Greifswald, Germany; 4 Department of Hand-, Replantation- and Microsurgery, BG Klinikum Unfallkrankenhaus Berlin, Warener Str. 7, 12683 Berlin, Germany

**Keywords:** vascularized bone graft, palmar radiocarpal artery, dorsal carpal branch of the ulnar artery, anterior interosseous artery, superselective angiography

## Abstract

*Background:* This study assesses the variability of the palmar radiocarpal artery (PRCA), dorsal carpal branch of the ulnar artery (DCBUA), and anterior interosseous artery (AIA) in superselective catheter angiographies of the wrist (SCAW). *Methods:* Secondary analysis of consecutive SCAW (2009–2011). Measurements of the distances of the PRCA to the midface of the radiocarpal joint, the DCBUA to the styloid process of the ulnar, and maximum diameters of PRCA, DCBUA, and AIA. *Results:* Seven female and ten male patients (mean 35 years) received SCAW. All patients suffered from Kienbock’s disease. The mean distance from the PRCA to the radiocarpal joint was 7.9 ± 2.3 mm and the distance from the DCBUA to the styloid process of the ulna was 29.6 ± 13.6 mm. The mean maximum diameter of the PRCA was 0.6 ± 0.2 mm, that of the DCBUA was 1.1 ± 0.4 mm, and that of the AIA 1.2 ± 0.3 mm. In six cases (35%), all three arteries contributed to the PRCA; in eight cases (47%), the radial and AIA; in two cases (12%), the radial and ulnar artery; and in one case (6%), only the radial artery contributed. *Conclusions:* SCAW are feasible to assist in preoperative planning. Os pisiforme transfer with DCBUA might be the best choice for a vascular bone graft in Kienbock’s disease.

## 1. Introduction

The macroscopic and microscopic anatomies of the carpus have mainly been studied intraoperatively or in wrist cadavers. Between 1980 and 2011, French groups published fundamental findings on the vascular anatomy of the wrist and offered an option for vascularized bone grafts using the palmar radiocarpal artery (PRCA) to approach scaphoid non-union and Kienbock’s disease [1,2,3,4,5,6]. Early studies showed that there is variability in the occurrence, length, and diameter of this artery. They described how the palmar radiocarpal arch normally arises between 1 and 2.5 cm proximal to the radial styloid and between 5 and 8 mm proximal to the distal radioulnar joint when the PRCA originates from the radial artery. The vessel passes horizontally along the distal border of the pronator quadratus muscle to form an anastomosis with the anterior interosseous artery (AIA) in a T-shaped fashion. Further horizontally, this anastomosis joins the ulnar artery to complete the palmar arch [1,7]. Gelberman et al. found that, while the palmar radiocarpal arch was present in 100% of their cadavers, it was formed by all three arteries in 87%, and in 13% solely by the radial and ulnar arteries. The diameter of the PRCA varied from 0.5 to 1 mm [8]. Before the first reports about bone grafts using the PRCA were published, Beck described the method of vascularized Os pisiforme transfer in patients with Kienbock’s disease [9]. This method utilizes the dorsal carpal branch of the ulnar artery (DCBUA) to harvest the vascularized Os pisiforme. Oppikofer et al. reported that this branch was present in 100% of studied cadavers and originated 1.6 to 4.4 cm proximally to the ulnar styloid, passing to the ulnar border of the wrist. The DCBUA was found in close proximity to the dorsal sensory branch of the ulnar nerve [4], and its diameter was measured to be 1.3 mm (+/− 0.2 mm) [10]. Yet another approach for vascular bone grafts is a bony pedicle of the distal radius harvested with branches of the AIA [11,12,13,14]. The AIA arises from the common interosseous artery, travels distally to the interosseous membrane, and anastomoses with the PRCA and the posterior interosseous artery [15]. The diameter of the AIA varied from 0.9 to 1.5 mm in published cadaver studies [16,17].

In the study center, SCAW were used in diagnostics and preoperative planning of specific cases, one indication being cases with advanced stages of Kienbock’s disease. In these instances, SCAW served to determine the blood flow to the lunate by the AIA, radial, and ulnar arteries in resting position and during wrist motion. During routine clinical practice, wrist surgeons are also recurrently faced with the challenge of evaluating possibilities for vascularized bone grafts in complex cases. Publication of our primary work [18] triggered the hope for a reproduciable imaging tool to measure the peripheral arteries of the wrist as another application of SCAW.

Kienbock’s disease is characterized by a vascular necrosis of the lunate [19]. Its etiology has long been debated in various studies and reviews of this pathology [19,20,21]. Most authors agree on a multifactorial genesis, including vascular aspects that promote infarction of the lunate through emboli, trauma, or vasculitis [19,20,22]. Therapeutic options for symptomatic Kienbock’s disease consist of conservative treatment, lunate decompression, and rarely vascularized bone grafts. Advanced stages of Kienbock’s disease have been approached by lunate replacement, wrist fusion, proximal row carpectomy, and wrist arthroplasty [23].

The following study assessed the variability of the PRCA, the DCBUA, and the AIA in superselective catheter angiographies of the wrist as proof of concept.

## 2. Materials and Methods

### 2.1. Protocol

We performed a secondary analysis of the study described in “*Superselective angiography of the wrist in patients with Kienbock’s disease*” [18].

The study was conducted in accordance with the Declaration of Helsinki of 2012 and approved by the institutional ethics committee of the University of Greifswald, Germany (BB 054/17). Informed consent was obtained for use of data and images prior to inclusion in the study from all patients.

**Figure 1:**
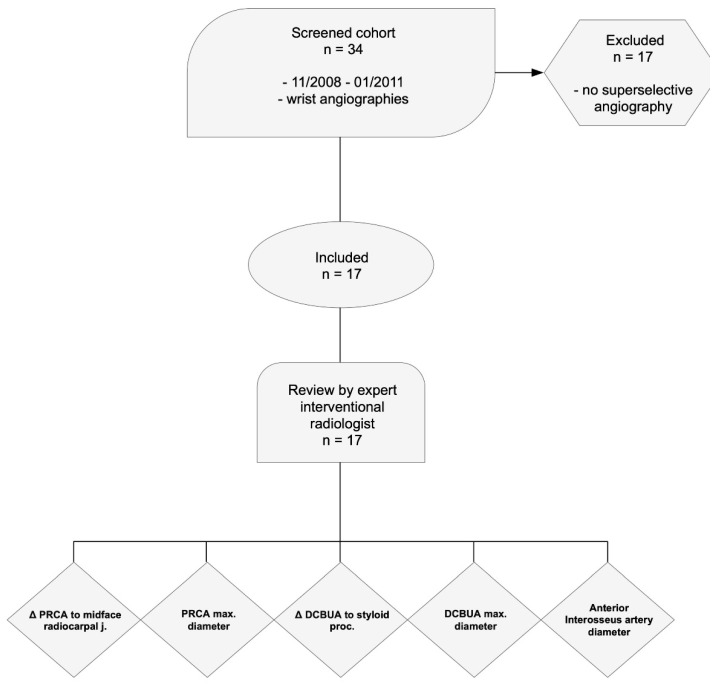
Study protocol according to the STROBE Initiative [24] showing patient screening, inclusion, exclusion, and analysis (STROBE = The Strengthening the Reporting of Observational Studies in Epidemiology Initiative; PRCA = palmar radiocarpal arch; DCBUA = dorsal carpal branch of the ulnar artery).

Between November 2008 and January 2011, the picture archiving and communication system was screened for SCAW at a large metropolitan trauma center with dedicated hand and wrist surgery. All consecutive cases were identified and included in the study retrospectively. Non-superselective wrist angiographies were excluded from the study (Figure 1).

### 2.2. Patients

All patients presented with Kienbock’s disease stages 2–4 on MRI according to the Lichtmann classification and were scheduled for surgical treatment [25]. Preoperative MRI was searched for narrowing of the ulnocarpal joint and direct signs of existing cartilaginous damage of the lunate fossa. Neutral, negative, and positive ulnar variances were determined.

None of the patients underwent surgery before the presentation. Catheter angiographies were performed to determine which surgical approach would endanger the vascular supply of the lunate the least. Additionally, the knowledge of compromised perfusion of the lunate in different wrist positions or the total lack of osseous perfusion of the lunate on SCAW was expected to influence individual therapeutic approaches in addition to MRI results and clinical presentation. Catheter angiographies were performed solely on the affected wrist to minimize the risk of intraprocedural complications and to reduce radiation exposure.

### 2.3. Superselective Catheter Angiographies of the Wrist (SCAW)

SCAW were conducted in the supine position with the arm and wrist in a neutral position, under general anesthesia to avoid vasoconstriction of peripheral vessels. Through a right femoral approach with a short 4F sheath, a 4F vertebral catheter was placed in the axillary artery of the affected arm. The first selective angiographic runs showed an overview of the angiographic anatomy of each patient. Then, a 170 cm long 0.0021″ microcatheter (Rapidtransit^®^, Codmann, Johnsons & Johnson, New Brunswick, NJ, USA) was inserted into the radial, ulnar, and common interosseous arteries for superselective angiographies in palmo-dorsal and lateral projections in resting position, flexion/extension, and radial-/ulnarduction. Angiographies were obtained using a flat panel digital subtraction angiography imager (Allura XPER FD 20/20, Philips Medical Systems, Amsterdam, The Netherlands).

### 2.4. Radiological Assessment

The maximum length of the Os pisiforme was measured on pre-interventional radiographs and served as a benchmark for calibration of the selective and superselective angiographies in palmo-dorsal projections.

**Figure 2:**
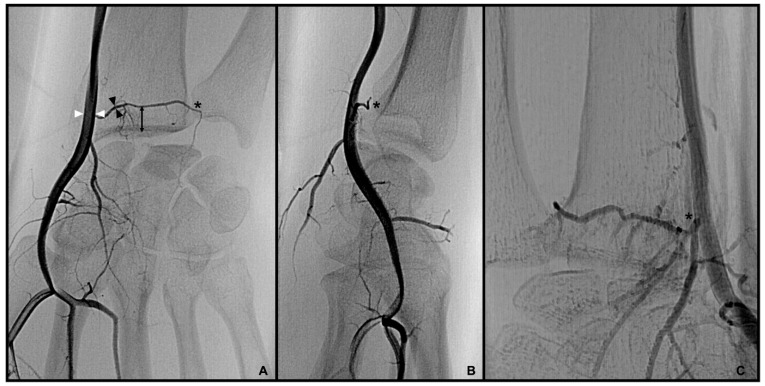
(**A**): Palmo-dorsal superselective angiography of the radial artery with the PRCA arising from one main branch. (**B**): Lateral projection of the radial artery to verify the palmar location of the vessel. (**C**). Palmo-dorsal superselective angiography of the radial artery with the PRCA arising as part of a ramification. * PRCA, ▲ maximum diameter of the PRCA, △ diameter of the radial artery at the point of ramification, ⟷ distance from the PRCA to the midface of the radiocarpal joint (PRCA = palmar radiocarpal arch).

**Figure 3:**
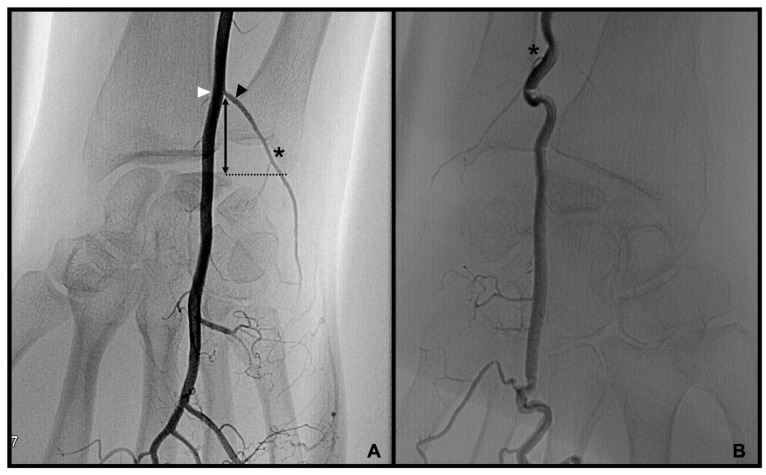
Palmo-dorsal superselective angiographies of the ulnar artery with a strong DCBUA (**A**) and a delicate DCBUA (**B**). * DCBUA, ▲ maximum diameter of the DCBUA, △ diameter of the ulnar artery at the point of ramification, 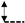 distance from the DCBUA to the styloid process of the ulnar (DCBUA = dorsal carpal branch of the ulnar artery).

Palmo-dorsal projection angiographies were used to identify the PRCA and the DCBUA (Figure 2A and Figure 3). Lateral projections were used to ensure the palmar location of the vessel identified as PRCA (Figure 2B). For topographic classification, the distance from the PRCA to the midface of the radiocarpal joint and the distance from the DCBUA to the styloid process of the ulna were measured in mm (Figure 2A and Figure 3A).

**Figure 4:**
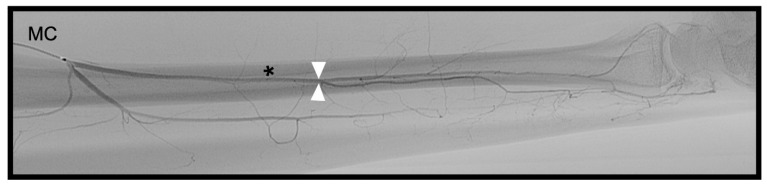
Palmo-dorsal superselective angiography of the * AIA. △ maximum diameter proximally to its main branches, MC inside the common interosseous artery (AIA = anterior interosseous artery; MC = microcatheter).

The maximum diameters of the PRCA and the DCBUA were measured in mm on superselective angiographies (Figure 2A and Figure 3). Both diameters were then related to the diameter of the main vessel, the radial, and ulnar artery, at the point of ramification, to account for smaller vessels in shorter or female patients (Figure 2A and Figure 3). It was determined if the PRCA was a main branch or part of a ramification (Figure 2A,C). On superselective angiographies, the contribution of the radial, ulnar, and AIA to the palmar radiocarpal arch was assessed, and the primary direction of the flow inside the arch was objectified. The diameter of the AIA was measured proximally to its main branches at the distal forearm in mm (Figure 4).

### 2.5. Statistical Analysis

Median diameters with standard deviations of the PRCA, the DCBUA, and the AIA, as well as the distances from the PRCA and the DCBUA to the midface of the radiocarpal joint and the styloid process of the ulna, respectively, were calculated using Microsoft Excel 2016 (^®^Microsoft) for iOS. The diameters of the PRCA and the DCBUA were related to their main vessels and expressed as

(1) $$\frac{diameter\;of\;PRCA\;or\;DCBUA}{diameter\;of\;main\;vessel}$$

Measurements were tested for normality. If a normal distribution of the values could be assumed, comparisons between the two groups were conducted using a two-sample *t*-test. The Mann–Whitney U test was applied if a nonparametric distribution of the measurements was suspected. The level of significance for multiple comparisons was adapted using the Bonferroni correction for *p*-values ≤ 0.05. GraphPad Prism 9 for Mac (GraphPad, San Diego, CA, USA) was used for statistical analysis.

## 3. Results

**Figure 5:**
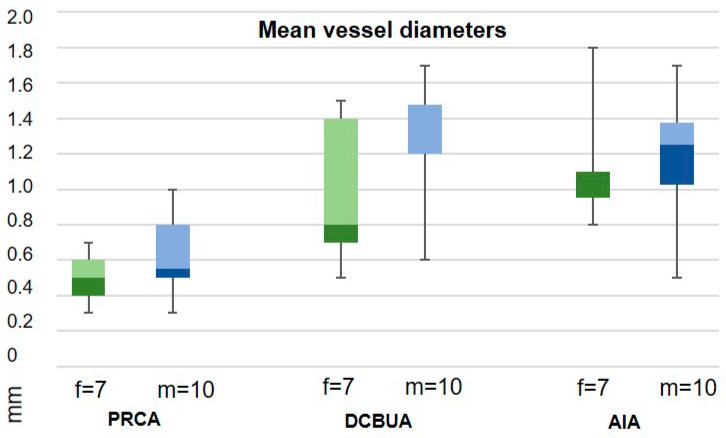
Mean vessel diameters of the palmar radiocarpal arch (PRCA), the dorsal carpal branch of the ulnar artery (DCBUA), and the interosseous artery (AIA) did not differ significantly between men (m) and women (f). Box plots with min/max values, median 25th and 75th percentile.

**Figure 6:**
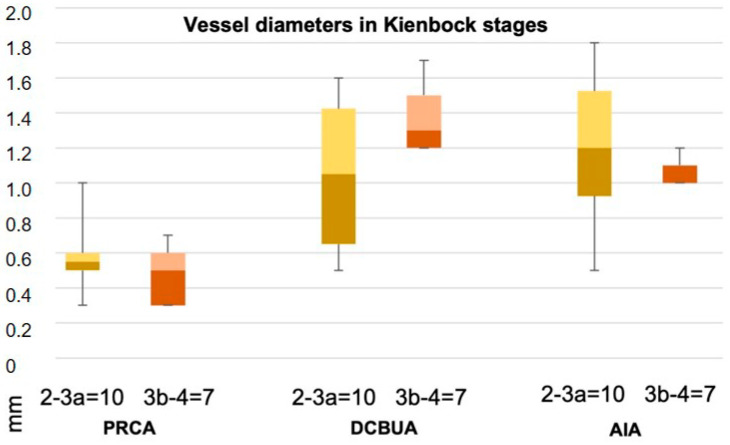
Mean vessel diameters of the palmar radiocarpal arch (PRCA), the dorsal carpal branch of the ulnar artery (DCBUA), and the anterior interosseous artery (AIA) in Kienbock’s disease stages 2–3a versus 3b did not differ significantly. Box plots with min/max values, median 25th and 75th percentile.

Seven female and ten male patients with a mean age of 35 years (median 31, range 19–64) were examined. Kienbock’s disease was located in the right hand in ten cases and in the left hand in seven cases. The dominant hand was affected in 47% of cases. The negative ulnar variance was present in thirteen cases, and in four cases a neutral position of the ulna was observed. Table 1 shows the measured parameters for each patient. The comparison of male and female patients showed no significant difference for all values (Table 1, Figure 5). The results of this study also did not show a statistically significant influence of Kienbock’s disease stage on vessel diameters (Figure 6).

### 3.1. Palmar Radiocarpal Artery and Palmar Radiocarpal Arch (PRCA)

**Figure 7:**
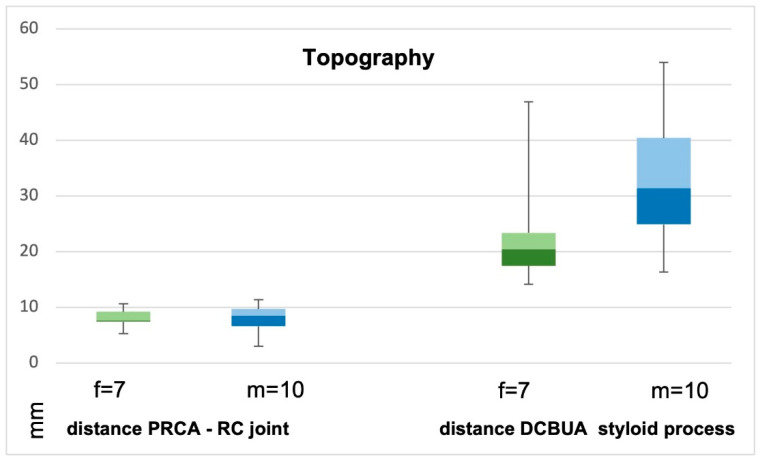
Distances from the palmar radiocarpal arch (PRCA) to the radiocarpal joint and from the dorsal carpal branch of the ulnar artery (DCBUA) to the styloid process of the ulna did not differ significantly between men (m) and women (f). Box plots with min/max values, median 25th and 75th percentile.

**Figure 8:**
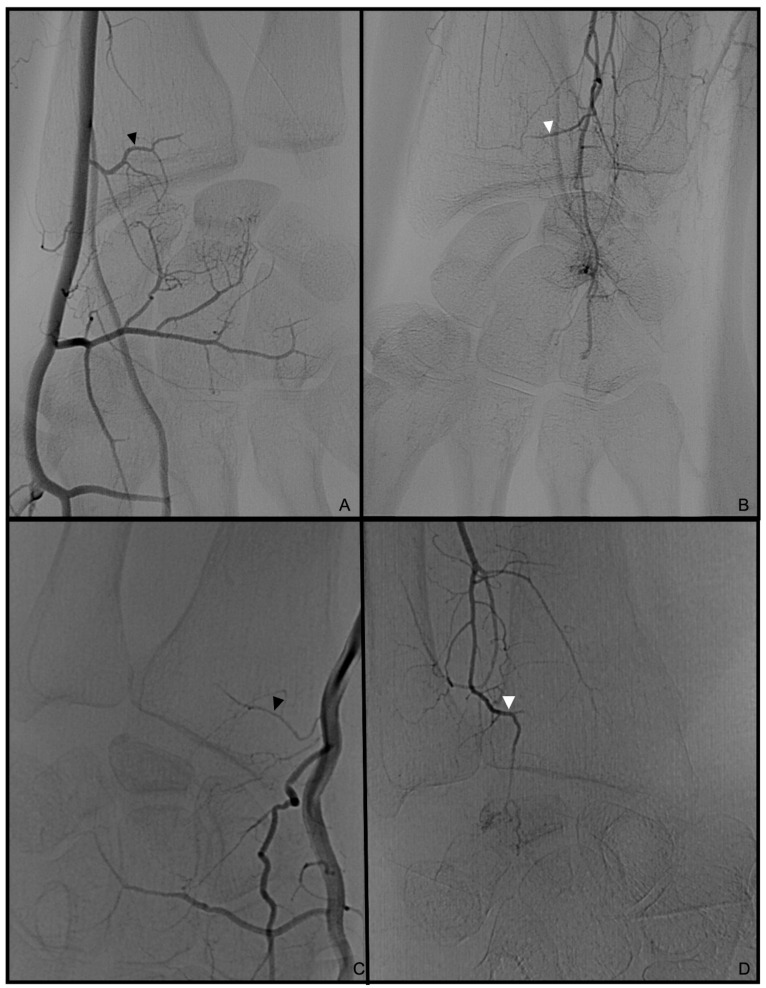
Palmo-dorsal superselective angiographies of the wrist. (**A**,**B**): Prominent palmar radiocarpal arch with strong PRCA and AIA. (**C**,**D**): The PRCA is below average in diameter, and the anastomosing branch of the AIA is not compensating for the lower flow of contrast agent. ▲ PRCA, △ anastomosing branch of the AIA (PRCA = palmar radiocarpal arch; AIA = anterior interosseous artery).

**Figure 9:**
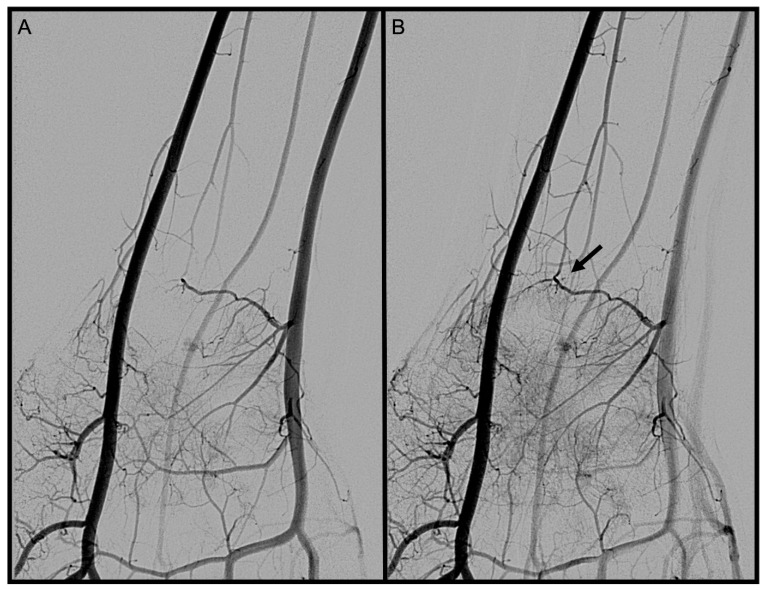
Palmo-dorsal angiography run of the wrist. (**A**): PRCA before anastomosis becomes visible. (**B**): The 🠪 shows the “L-shaped” anastomosis between the PRCA and the AIA (PRCA = palmar radiocarpal arch; AIA = anterior interosseous artery).

The PRCA was identified in all selective angiographies. In twelve patients, the PRCA arose as one main branch and, in five cases, it arose as part of a ramification of several branches. The mean distance from the PRCA to the midface of the radiocarpal joint was 7.9 mm ± 2.3 mm (range 3–11.4 mm). This distance did not differ significantly between the sexes (*p* = 0.86) (Figure 7). The mean maximum diameter of the PRCA was 0.6 mm ± 0.2 mm. The PRCA measured ≦0.5 mm in nine cases and ≧0.6 mm in eight cases (range 0.3–1 mm) (Figure 8). The relative diameter of the radial artery at the point of ramification to the PRCA ranged between 0.14 and 0.33 (mean 0.24). The contribution of the radial, ulnar, and AIA to the palmar radiocarpal arch was examined closely on superselective angiographies. In six cases (35%), all three arteries contributed to the perfusion of the palmar radiocarpal arch; in eight cases (47%), the radial and AIA contributed to the perfusion (Figure 9); in two cases (12%), the radial and ulnar arteries contributed; and in one case (6%), the palmar radiocarpal arch was only perfused through the radial artery. The primary direction of the contrast agent flow inside the arch was noted from the radial artery in 14 cases. In two cases, the flow of the contrast agent seemed evenly distributed from the radial and ulnar arteries and in one case from the radial and AIA.

### 3.2. Dorsal Carpal Branch of the Ulnar Artery (DCBUA)

The DCBUA was identified in all selective angiographies. The mean distance from the DCBUA to the midface of the styloid process of the ulna was 29.6 mm ± 13.6 mm (range 14.1–56.9 mm). This distance was insignificantly greater in men than in women (32.97 mm ± 12.56 mm vs. 24.73 mm ± 14.59 mm, *p* = 0.23) (Figure 7). The mean maximum diameter of the DCBUA was 1.1 mm ± 0.4 mm (range 0.5–1.7 mm). In six patients with a small PRCA of ≦0.5 mm, the DCBUA showed a diameter of >1.1 mm. The relative diameter of the ulnar artery at the point of ramification to the DCBUA ranged between 0.22 and 0.65 (mean 0.43).

### 3.3. Anterior Interosseous Artery (AIA)

The AIA was identifiable in all cases and anastomosed with the PRCA (Figure 9). The mean diameter of the AIA proximally to its main branches at the distal forearm was 1.2 mm ± 0.3 mm (range 0.5–1.8 mm).

### 3.4. Treatment Strategies

For a detailed description focusing on the angiographic perfusion of the lunate, we refer to our previous study [18]. The blood supply to the lunate was reduced or even discontinued in 12 patients during extension/flexion and/or radial-/ulnarduction. In five patients, no relevant perfusion of the lunate was observed. In eight out of twelve cases with reduced blood flow during forced wrist motion, lunate decompression by radial shortening osteotomy was indicated to decelerate the progression of the disease. A negative ulnar variance was present in all 12 cases. In the four other cases, severe cartilaginous damage was suspected on MRI and confirmed during arthroscopy, which is why they were treated by synovectomy only. The remaining five patients without any angiographic perfusion of the lunate also received solely synovectomy, because it was anticipated that completely disrupted blood flow could not be restored. In this latter group, proximal row carpectomy was performed in four patients during the further course of treatment due to persistent pronounced wrist pain.

After radial shortening, osteotomy consolidation of the lunate was observed in five out of eight cases. Another patient developed severe cartilaginous damage and received secondary synovectomy and selective denervation. Two patients were lost to follow-up.

## 4. Discussion

The results of this study show a novel approach for preoperative planning: the calibers, origin, and flow directions of the PRCA and the DCBUA can be evaluated on superselective catheter angiographies.

In the present study, the PRCA was detectable in all 17 cases, but in only 35% of the cases, a contribution of the radial, ulnar, and AIA, and in 12% of the radial and ulnar arteries was observed angiographically. In contrast, a cadaver study reported that, in 87% of cases, all three arteries anastomosed to the palmar radiocarpal arch, whereas in 13%, only the radial and ulnar arteries formed the arch [7]. The AIA was not mentioned in this classification. Another cadaver study described that an anastomosis of the AIA occurred in all dissections [2]. Superselective angiographies revealed that the AIA anastomosed frequently (47% of the cases) with the palmar radiocarpal arch. It could be hypothesized that the branch of the AIA to the palmar radiocarpal arch is often quite delicate, which might hinder the detection in cadaver studies and angiographies. This assumption is supported by the primary direction of the contrast agent flow on superselective angiographies inside the arch, which was detected to originate from the radial artery in the majority of cases. Haerle et al. also reported that the PRCA dominantly contributes to the palmar radiocarpal arch [4]. Furthermore, the present study investigated the proximal anatomy of the PRCA. The authors imagined that the diameter of the vessel could be influenced if the vessel arose as part of a ramification of several branches. Nonetheless, the results of this study could not support this hypothesis. Moreover, angiographic and anatomical results confirm that the PRCA sometimes passes the midface of the radiocarpal joint horizontally in close proximation of only 3–5 mm, which allows only for the preparation of a small pedicle [2,7]. If assessed correctly in advance or intraoperatively, surgeons could vary their approach from the “T-shaped” point of anastomosis to the PRCA and harvest an adequate bone graft laterally [1].

The angiographically measured diameters of the PRCA (0.3–1 mm) compared well with the cadaver studies of Gelbermann et al. and Haerle et al. [4,8]. In both studies, the PRCA measured less than 0.5 mm in only one case out of sixty-five, whereas the angiographic results revealed four cases out of seventeen in which the PCRA was thinner than 0.5 mm. It should be noted that neither cadaver study mentioned the distribution of male vs. female wrists in their collectives. To address the issue of a dependency of vessel diameter on gender, the present study included binary relations for all cases. However, the vessel diameters did not differ significantly between the sexes for PCRA, DCBUA, and AIA.

The DCBUA has rarely been described in detail in the current literature. Oppikofer et al. showed that this vessel was present in 100% of dissected wrists. The mean diameter of the DCBUA of 1.3 mm ± 0.2 mm was similar to the angiographic result of 1.1 mm ± 0.4 mm. In 12 cadaveric wrists, the DCBUA originated 1.6 to 4.4 cm proximally to the ulnar styloid [10]. The current angiographic study showed an even wider range varying from 1.4 to 5.7 cm. Measurements of the proximal AIA on SCAW (range 0.5–1.8, mean 1.2 mm ± 0.3 mm) were similar to the DCBUA and lay within the range of published cadaver studies that described mean calibers of 1.28 mm and 0.9–1.5 mm [16,17].

The results of this secondary analysis summarize the two clinical applications of SCAW: 1. Functional information about the perfusion of the carpus [18] can support therapeutic decisions aimed at improving blood supply, e.g., to the lunate-by-lunate decompression; and 2. detailed, revisable, and reproducible knowledge of individual anatomy and vessel diameters can facilitate operative planning and execution of vascularized bone grafting. Unlike ultrasound and MRI studies, catheter angiographies are invasive procedures. However, despite ongoing progress in radiological imaging, catheter angiographies are still referred to as the most precise technique for imaging delicate vasculatures [26,27].

In Kienbock’s disease, the blood supply of the lunate bone seemed to be less dependent on the DCBUA than PRCA and AIA in cadaver studies and our previous report [18,28]. The slightly greater vessel diameter of the DCBUA compared to the PRCA in the literature suggests that transferring the Os pisiforme with DCBUA might be the most reliable choice for a vascular bone graft in Kienbock’s disease, with a smaller risk of further worsening the blood supply of the lunate by altering the similarly strong AIA.

Certain limitations of this study must be addressed. First, this retrospective study merely includes the description of the anatomies of the 17 cases. A greater number of patients would increase the consistency of the results, reduce the standard deviations of the measurements, and confirm the statistical analysis. However, substantiated indications for wrist angiographies are rare, as long as the benefit of preoperative angiographies remains uncertain. Second, the study lacks a comparison with a healthy control group. Although no significant difference between the vessel diameter in Kienbock’s disease stages 2–3a compared to stages 3b–4 was observed, all patients suffered from clinically apparent disease and sought medical help. Catheter angiographies of healthy patients or bilateral angiographies could enable the transferability of the results to other patients, but these additional angiographies cannot be medically justified due to increased radiation exposure and potential procedural risks. Nevertheless, the current study might serve as a proof of concept and pave the way for studies on further application of SCAW in preoperative imaging in the future.

## 5. Conclusions

In addition to detailed information about the blood supply to the carpus, superselective catheter angiographies provide precise measures of the calibers of peripheral arteries of the wrist. Evaluation of the calibers, origin, and flow directions of PRCA, DCBUA, and AIA seems practical, and could assist in preoperative planning and decision making before vascularized bone grafts of the carpus. In Kienbock’s disease, transferring the Os pisiforme with DCBUA might be the more reliable choice for a vascular bone graft.

## Figures and Tables

**Table 1:** Vessel diameters of 17 cases on superselective catheter angiographies.

Patient	Gender	Disease Stage on MRI	Δ PRCA to Midface Radiocarpal j. (mm)	PRCA Max. Diameter (mm)	Relation RA to PRCA	Δ DCBUA to Styloid Proc. (mm)	DCBUA Max. Diameter (mm)	Relation UA to DCBUA	AIA Max. Diameter (mm)
1	M	2	4.1	0.4	0.25	18.5	0.6	0.22	0.5
2	M	3a	9.3	***1.0***	0.32	54.0	0.9	0.32	1.7
3	M	3a	6.6	0.5	0.14	25.2	***1.5***	0.38	1.3
4	M	3a	3.0	0.5	0.2	31.6	***1.2***	0.56	1.3
5	M	3a	11.4	***0.8***	0.33	41.4	***1.2***	0.33	1.6
6	M	3a	10.6	***0.6***	0.19	49.3	***1.6***	0.45	1.0
7	M	3b	6.5	0.5	0.2	16.3	***1.2***	0.65	1.0
8	M	3b	9.6	***0.8***	0.29	31.2	***1.4***	0.43	1.4
9	M	3b	7.6	0.3	0.15	24.8	***1.7***	0.64	1.1
10	M	4	9.7	***0.8***	0.3	37.4	***1.2***	0.37	1.2
11	F	2	7.5	***0.6***	0.22	14.1	0.8	0.54	1.1
12	F	3a	5.3	0.3	0.16	16.9	0.6	0.28	0.8
13	F	3a	7.3	0.5	0.23	20.4	0.5	0.28	0.9
14	F	3a	10.0	***0.6***	0.24	18.0	***1.5***	0.45	1.8
15	F	3b	7.7	0.5	0.26	24.2	0.8	0.5	1.1
16	F	3b	8.4	***0.7***	0.3	22.6	***1.3***	0.43	1.1
17	F	3b	10.1	0.3	0.21	56.9	***1.5***	0.45	1.0
*p*-value	F vs. M		0.96	0.36	0.96	0.11	0.27	1.0	0.32
*p*-value	2/3a vs. 3b/4			0.88			0.27		0.84

Measurements of all vessel diameters on superselective angiographies with *p*-values for comparisons of men vs. women, and of stages 2/3a vs. 3b/4 of Kienbock’s disease. M = male, F = female, Δ = distance, RA = radial artery, UA = ulnar artery, bold and italics = vessel diameter exceeds mean.

## Data Availability

All data are contained within the article.

## Abbreviations

SCAW	superselective catheter angiographies of the wrist
PRCA	palmar radiocarpal artery
DCBUA	dorsal carpal branch of the ulnar artery
AIA	anterior interosseous artery
STROBE	The Strengthening the Reporting of Observational Studies in Epidemiology Initiative
